# The freedom of choice

**DOI:** 10.1186/s40694-016-0027-5

**Published:** 2016-10-14

**Authors:** Vera Meyer, Alexander Idnurm

**Affiliations:** 1 Institute of Biotechnology, Berlin University of Technology, Berlin, 13355 Germany; 2 School of BioSciences, The University of Melbourne, Parkville, VIC 3010 Australia

In our welcome editorial to launch *Fungal Biology and Biotechnology* in October 2014 we stated that ‘This is a new golden age of discovery in the fungi, and an exciting time to take part of this adventure’ [1]. At that time we made a number of promises and predictions (Box 1). Now, two years after the birth of the first open-access journal devoted to fungal bio(techno)logy it is time to take stock of what has been achieved and what further progress needs to be made on making the vision and promise for this exciting journal to come true.

**Box 1:** Statement from opening editorial

We intend for the journal to become a hub for researchers seeking information on their favorite topics as well as considering new or alternative directions. The journal shall become a platform for scientists from academia and industry to present their hottest findings in unicellular or multicellular fungal systems, in medical or industrial strains, and in so far unexplored species. This will be a platform for experts to discuss their visions on how fungi can help us to address some of the key challenges of the twenty-first century [1]	

Let’s look at the facts: 26 peer-reviewed articles have been published from 51 manuscripts submitted to *FBBiotech* within the last two years. This selectivity reflects a strong basis to publish articles that are scientifically valid and feature new directions, and also on our wish to publish articles that are of interest to the fungal research community. These articles have the potential to shape future directions within the sciences of fungal bio(techno)logy—because they open up new avenues, represent considerable scientific or technological breakthroughs and/or ‘think’ outside the box. Indeed, the articles published by *FBBiotech* have become a success story: in total, more than 62,000 article accesses can be counted with an average of 2600 accesses per article (as of September 22nd, 2016), which compares favorably with other fungal articles published in well-established BMC journals in the field of applied microbiology from the same period (October 2014–September 2016) such as *Microbial Cell Factories* (1000 accesses/article), *BMC Microbiology* (1400 accesses/article) and *Biotechnology for Biofuels* (2200 accesses/article). The 2016 citations to papers published in 2014 and 2015, and with still a quarter of the year to go, already account for a current unofficial minimum journal impact factor (JIF) of 2.7, as identified through Google Scholar and Springer, and then each citation validated manually. We will be in contact with Thomson Reuters about being tracked for an official JIF, which is currently projected to be above 3, if calculated also for the citations for the 2016 papers. We thank the members of the editorial board [2], reviewers and authors for their support of this new journal. Without an investment by the research community, this level of success would not have been possible.

The majority of first or main authors of *FBBiotech* papers come from Germany (32 %), the US (14 %), The Netherlands (13 %) and UK (11 %) and the majority of readers come from the US (28 %), India (20 %), Germany (17 %), China and UK (each 10 %), thus from countries with strong fungal scientific communities and/or from countries with considerable public funding and private investment in the field of fungal biotechnology.

The open-access philosophy of *FBBiotech* generates high ‘scores’ for *FBBiotech* articles when alternative metric methods are evaluated as well. These methods, such as Altmetric [3], measure not only downloads and the citation frequency but also the attention surrounding an article. The most recent *FBBiotech* publications have especially generated a huge interest in the scientific and public community, e.g. with Altmetric scores of 7–11 putting them into the top 25 % of all research outputs scored by Altmetric. Manuscripts expected to be of high relevance to the fungal research community indeed became the most read, cited and discussed papers, and thus the most influential and in cases most inspirational ones. The three most read are Claudio Scazzocchio’s opening review that discusses his views on how fungal biology has changed during his career and particular in the post-genomic era [4], the Richter et al. research article on the new use of *Aspergillus niger* as a highly efficient production platform for secondary metabolites [5], and the research article of Matsu-ura et al. demonstrating the successful establishment of the CRISPR-Cas technology for genome editing purposes in *Neurospora crassa* [6].

Beyond the conventional article, *FBBiotech* aimed to address other aspects of how fungi impact society. Two commentaries are worth noting. First, Nai et al. reflect on the social responsibilities as scientists to talk more about our research on microorganisms as well as the need for new technologies to enable the microorganisms themselves to do their own ‘talking’ [7]. Second, the results of the first European academic-industry Think Tank meeting held ever in the history of fungal science was just published as a White Paper in *FBBiotech* [8]. This timely and foreword looking paper discusses research opportunities and challenges in fungal biology and biotechnology for the coming decade and defines the roadmap on how better to exploit industrially-used fungi and more efficiently fight pathogenic fungi, and thus make us fungal scientists significantly contribute to the world-wide manifold efforts essential to secure human welfare in the twenty-first century.

Using the online format of the journal also opens other opportunities to promote fungal biology more widely. *FBBiotech* has sponsored poster prizes at the different fungal conferences, including the European Fungal Genetics Conference held this year in Paris (ECFG13). Three posters of young scientists were selected in three sessions as being highlights, and the authors then interviewed about their work and visions, with this information posted on the BioMed Central blog and linked to the *FBBiotech* homepage. The journal will again feature at the 29th Fungal Genetics Conference at Asilomar Conference Center in March 2017.

Taken together, the journal *FBBiotech* has established itself—although still in its infancy—as an important platform to communicate key findings within the scientific community. The journal also provides an important multiplying factor by offering scientists the opportunity to communicate their findings across sectors as well as to the public, and by this generating considerable interest for their own work in a broader framework.

Our opening editorial also touched on concerns in the publishing world that had become evident, particularly the rise of predatory journals. A new issue is at the other extreme, with how the reputation of well-established journals is being misused to infer the value of scientific contributions by individual researchers. Recent initiatives explore ways out of the “preoccupation of many scientists with publishing their work in a journal with the highest impact factor […] with given clinical names such as ‘journal mania’, ‘IF mania’ and ‘impactitis’” [9]. The American Society for Microbiology has thus decided this year to remove journal impact factor information from all ASM journal websites (see e.g. [10]). We congratulate the editors of these journals for their stand to fight against the inappropriate focus on JIFs and their recent misuse for publication, funding, hiring, and even promotion decisions [11–13]. As authors and editors we have the freedom to choose where we want to see our work published and which journals we consider as the most appropriate ones for communicating our research data, opinions and visions to our community. We are convinced that this decision should only be based on high-quality science, rigorous peer-reviewing by academic experts, a fast-handling process and—last but not least—an open-access policy. All of this is ensured by *FBBiotech*. We thus hope to handle your submissions soon!
